# Supplementary material for: Urinary Free Cortisol Determination and Interferences Studies Using Liquid Chromatography Coupled to Tandem Mass Spectrometry after On-Line Solid Phase Extraction Based on TurboflowTM Chromatography
Source: Metabolites. 2023 Oct 9;13(10):1063. doi: 10.3390/metabo13101063 (PMC10608966; doi:10.3390/metabo13101063)
Supplement: Supplementary file 1 [file metabolites-13-01063-s001.zip › metabolites-2616904-supplementary.pdf]

## Supplementary Materials

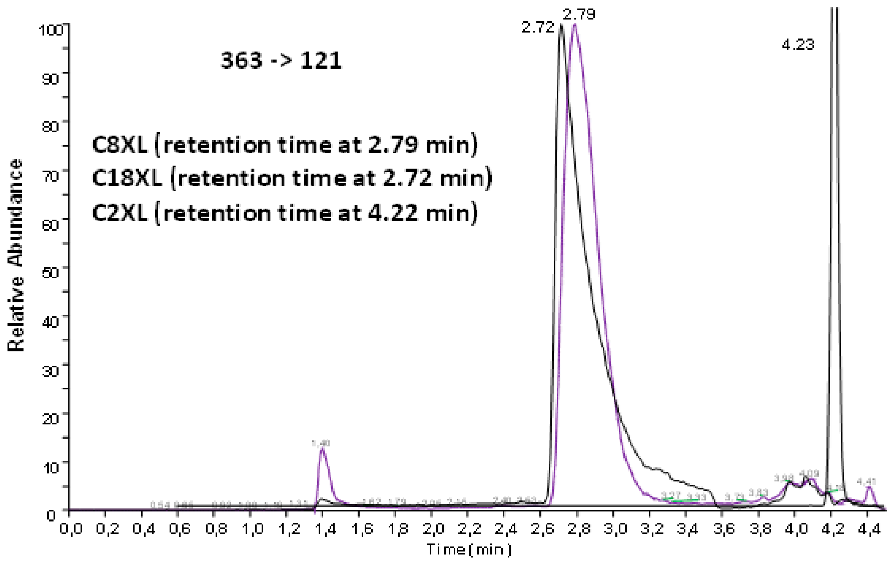


**Figure S1**: **Chromatograms obtained with C8XL, C18XL and C2XL extraction columns.** For C8XL and C18XLcolumns, the initial percentage of B1 used is 40%. For C2XL, the initial percentage of B1 used is 25%. The chromatogram obtained with C2XL is not at the same scale as the others.

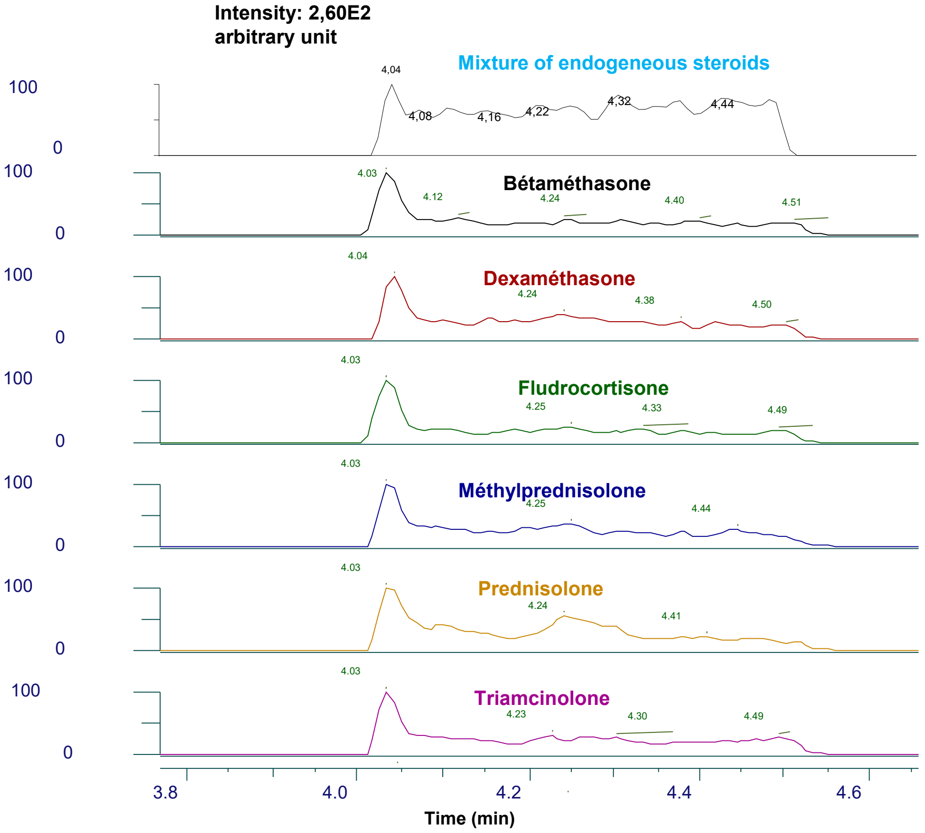


**Figure S2: Endogeneous and exogeneous steroids analysis by LC-MS/MS.** Chromatogramms (363->121 and 366->124) obtained by analyzing solutions of exogenous and endogenous steroids listed in the part 2.1, with the LC-MS/MS method described in 2.4 and 2.5 (cortisol analysis).

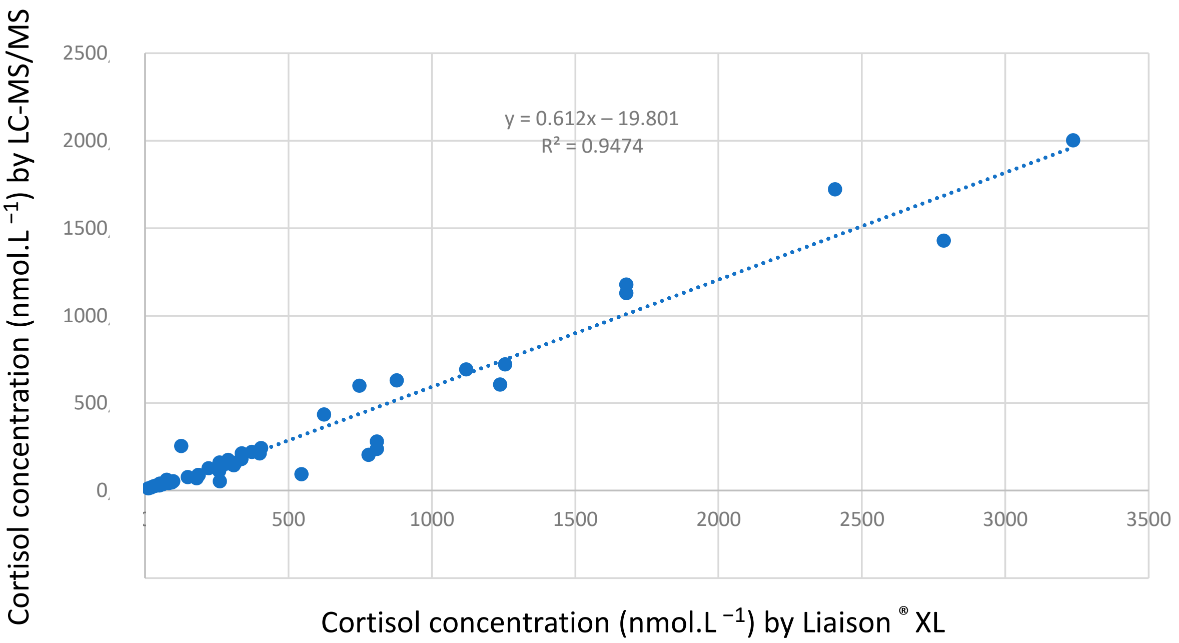


**Figure S3: Correlation between urinary free cortisol concentration values determined by LC-MS/MS and Liaison$^{R}$ XL** (n=47).
